# The Diagnostic Efficacy of Thrombelastography (TEG) in Patients with Preeclampsia and its Association with Blood Coagulation

**DOI:** 10.1515/biol-2019-0037

**Published:** 2019-07-22

**Authors:** He Lidan, Wu Jianbo, Gao Liqin, Hu Jifen, Lu Lin, Wu Xiuyan

**Affiliations:** 1 Department of Obstetrics and Gynecology, the First Affiliated Hospital of Fujian Medical University, Fuzhou Fuzhou Fujian Province 350005 PR China; 2 Department of Clinical laboratory, the First Affiliated Hospital of Fujian Medical University, Fuzhou Fuzhou Fujian Province 350005 PR China

**Keywords:** thrombelastography, preeclampsia, diagnosis, sensitivity, specificity

## Abstract

**Objective:**

The aim of this study was to investigate the diagnostic efficacy of thrombelastography (TEG) in patients with preeclampsia.

**Methods:**

One hundred and seventeen pregnant women were recruited from Department of Obstetrics and Gynecology of 1^st^ affiliated Hospital of Fujian Medical University. Of the 117 patients, 59 were normal late gestation (control group), 32 were mild preeclampsia and other 26 cases were severe preeclampsia. All the patients were received thrombelastography (including: K time, Reaction time, Clot angel, MA value, CI value) and blood coagulation examination (including: PT, APTT, Fib, TT, D-dimer and AT-III).

**Results:**

The R time, K time, Coagulation Index value and Clot Angle in preeclampsia group were significant different between control and preeclampsia groups with statistical difference (p<0.05). Moreover, the R and K time value in severe preeclampsia group were significant higher than those of control groups (p<0.05); however, the Coagulation Index value and Clot Angle in severe preeclampsia group were significant higher than those of mild preeclampsia group with statistical difference (p<0.05). Coagulation Index had the highest diagnostic sensitivity [87.93 (76.70-95.01) %] and specificity [83.83 (79.17-96.18)%] compared to other parameters with the AUC of 0.94 (0.90-0.98). The K time and the Coagulation Index had the highest diagnostic sensitivity (96.15%) and specificity (0.75%) respectively with the AUC of 0.68 and 0.75 respectively in differential diagnosis of severe preeclampsia from mild preeclampsia. However, there were no statistical difference in the aspects of platelet count and parameters relevant to coagulation test for the control, mild and sever preeclampsia groups(p>0.05).

**Conclusion:**

TEG provides more accurate information in monitoring the blood coagulation of preeclampsia patients and can be used as a reliable marker for assessing the severity of preeclampsia.

## 1 Introduction

The etiology of preeclampsia remains unclear. Currently, generally accepted theories assume that insufficient trophoblastic invasion causes shallow placenta implantation in the early stage of pregnancy. By the end stage of pregnancy, immune and placental factors cause systemic vascular endothelial cell disorder and dysfunction and the development of hypertension, proteinuria, blood hypercoagulability, and other clinical manifestations [1]. With the development of preeclampsia, coagulation factors and platelet consumption reduce, and local microthrombosis forms, leading to ischemia and hypoxia of important organs and the development of eclampsia, HELLP syndrome, disseminated intravascular coagulation (DIC), and multiple organ dysfunction syndrome (MODS), which seriously threaten the life of mother and child and primarily cause maternal and fetal death [2, 3, 4, 5].

Predicting the development of preeclampsia and judging the severity of the disease has consistently been a difficult problem in the field of obstetrics. However, thus far, no effective and reliable prediction methods are available. Several published studies have shown the degree of hypercoagulability in a patient is positively correlated with preeclampsia severity [6, 7, 8]. Therefore, monitoring the status of coagulation–fibrinolysis system in preeclampsia patients aids in evaluating the disease severity, whereas early clinical intervention can effectively improve the prognosis of mother and infant [9, 10, 11].

## 2 Material and methods

### 2.1 Patients

One hundred and seventeen pregnant women were recruited from Department of Obstetrics and Gynecology of 1^st^ affiliated Hospital of Fujian Medical University. All of the subjects provided written informed consent. Of the 117 patients, 59 were normal late gestation, 58 were mild preeclampsia and other 26 cases were severe preeclampsia. All patients were single pregnancy and the patients with diabetes, chronic hypertension, thrombocytopenia, thalassemia, severe liver, kidney diseases and autoimmune diseases were excluded.

**Informed consent**: Informed consent has been obtained from all individuals included in this study

**Ethical approval**: The research related to human use has been complied with all the relevant national regulations, institutional policies and in accordance the tenets of the Helsinki Declaration, and has been approved by the Medical Ethics Committee of 1st Affiliated Hospital of Fujian Medical University.

### 2.2 Blood sample collection

A total of 2 ml venous blood from the forearm elbow is obtained from all parturients within 12 h after admission to the hospital under the condition that no magnesium sulfate nor antihypertensive medicines are used. The blood sample is placed in 109 mmol/l sodium citrate 0.2 ml vacuum anticoagulation tube (9:1). The blood is sent for examination within 2 h after blood sampling, and Thrombelastography (TEG) test is carried out to measure the following: (1) coagulation time (R value); (2) coagulation rate (K value); (3) coagulation angle; (4) maximum amplitude; (5) coagulation comprehensive index (Fiure.1). At the same time, blood samples are obtained for routine blood coagulation function and blood routine examination.

### 2.3 Instruments and reagents

The study utilizes the following: CFMSTM TEG 5000 instrument (Haemonetics® Braintree, Massachusetts, USA ), kaolin activator, sodium citrate 0.2 mL vacuum anticoagulant tube (9:1), and 0.2 mmol/L calcium chloride test cup. The traditional coagulation function tests are carried out by SYMEX CS5100 instrument and its matching reagent. The platelets are counted by adopting the SIEMENS ADVIA 2120 instrument and the matching reagent.

### 2.4 TEG examination method

A total of 1 mL of blood sample is added into the kaolin activator bottle, which is gently inverted evenly for five times. Then, 340 μL of the inverted sample is added into a test cup with 20 μL of 0.2 mmol/L calcium chloride. In the previously mentioned instrument, the “Crited Kaolin” sample type is selected, the cup frame is raised, the lever rod is moved to the test position, and the test is started. The values of five TEG indexes are recorded according to the test results of the instrument [12, 13]. Prothrombin time, activated partial thromboplastin time, fibrinogen, and thrombin time are detected by coagulation. D-dimer is detected by immune turbidimetry. Antithrombin ^Ⅲ^ is detected by chromogenic substrate method. Platelet count is analyzed by whole-blood cells.

### 2.5 Statistical analysis

Data expressed as the mean±sd were analyzed by STATA11.0 software (http://www.stata.com; Stata Corporation, College Station, TX). The difference between controls and preeclampsia subjects was analyzed by one way ANOVA analysis. Diagnostic sensitivity and specificity was calculated by the equation of sensitivity=true positive/(true positive+ false negative), specificity=true negative/(true negative+ false positive). The area under the receiver operating characteristic (ROC) curve was used to evaluate the feasibility of TEG as reference for preeclampsia diagnosis. Two tailed p<0.05 was considered statistically significant.

## 3 Results

### 3.1 Parameters of TEG comparison

The R time, K time, Coagulation Index value and Clot Angle in preeclampsia group were significant different between control and preeclampsia group with statistical difference (p<0.05), **Table 1**. Moreover, the R and K value in severe preeclampsia group were significant higher than that of control groups (p<0.05); however, the CI value and Angle in severe preeclampsia group were significant higher than that of mild preeclampsia group with statistical difference (p<0.05), **Figure 2**.

**Figure 1:**
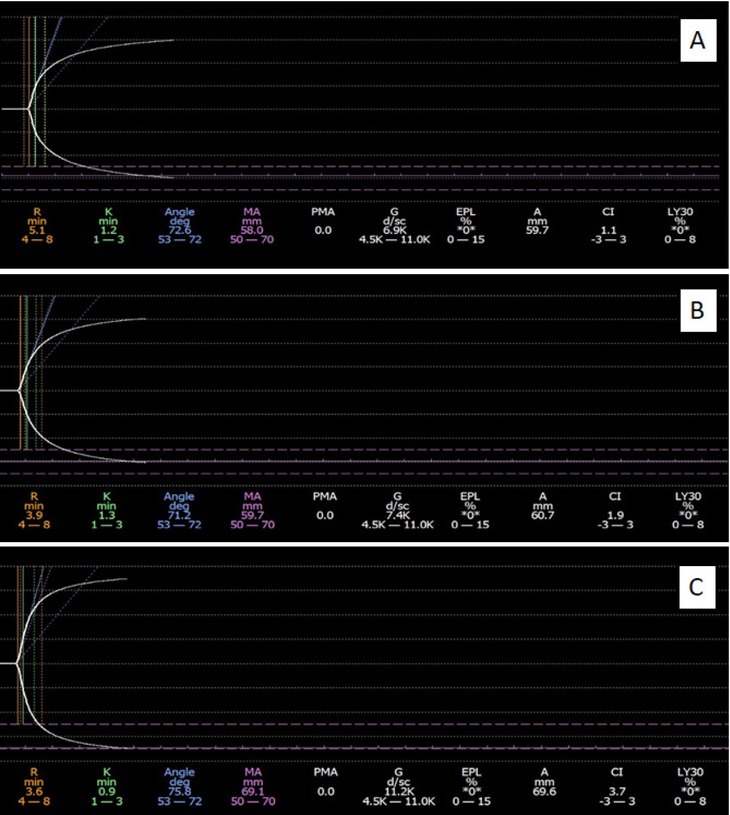
Thrombelastograghy examination for normal late gestation(A), mild preeclampsia(B) and severe preeclampsia(C).

**Figure 2:**
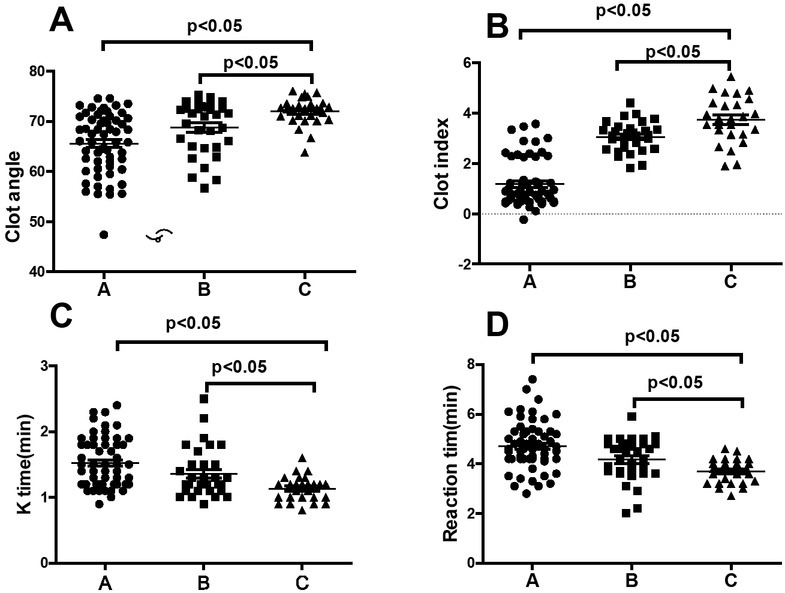
Scatter plot of clot angle, Coagulation Index, K time and reaction time distribution(A:Clot angle; B:Coagulation Index, C:K time; D:Reaction time)

**Table 1:** Parameters of thrombelastography comparison between control and preeclampsia groups

Groups	*_n_*.	R time (min)	K time (min)	Clot Angle (°)	Coagulation Index
Control	59	4.71±0.97	1.52±0.37	65.58±6.08	1.17±0.93
mild preeclampsia	32	4.17±0.86^*^	1.36±0.38^*^	68.76±5.27^*^	3.06±0.58^*^
severe preeclampsia	26	3.70±0.50^*#^	1.13±0.19^*#^	71.95±2.77^*#^	3.74±0.92^*#^

*compare to control group, p＜0.05；#：compared to mild preeclampsia，*P*＜0.05。

### 3.2 Diagnostic efficacy of TEG

**Figure 3:**
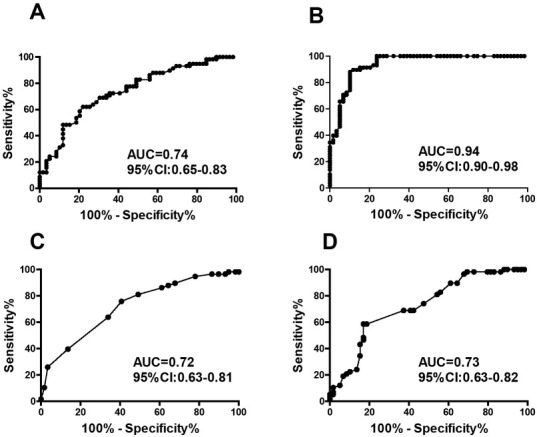
ROC curve of thrombelastograghy relevant parameters in diagnosis of preeclampsia (A:Clot angle; B:Coagulation Index, C:K time; D:Reaction time)

The diagnostic efficacy of parameter relevant to TEG were demonstrated in table 2. Coagulation Index had the highest diagnostic sensitivity [87.93 (76.70-95.01) %] and specificity [83.83 (79.17-96.18)%] compared to other parameters with the AUC of 0.94 (0.90-0.98), **Figure 3**.

**Table 2:** Efficacy of clot angle, Coagulation Index, K time and reaction time in diagnosis of preeclampsia

Index	Sen	Spe	AUC	Cut off
Clot Angle	72.41(59.10-83.34)%	64.41(50.87-76.45)%	0.74(0.65-0.83)	68.60
Coagulation Index	87.93(76.70-95.01) %	83.83(79.17-96.18)%	0.94(0.90-0.98)	2.47
K time	75.86(62.83-86.13)%	59.32(45.75-71.9)%	0.72(0.63-0.81)	1.35
R time	58.62(44.93-71.40)%	81.36(69.09-90.31)%	0.73(0.63-0.82)	3.14

### 3.3 TEG relevant parameters in differential diagnosis of severe preeclampsia

**Figure 4:**
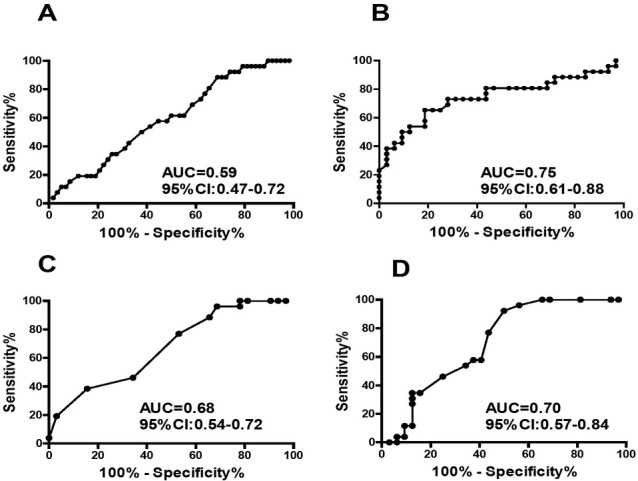
ROC curve of TEG relevant parameters in differential diagnosis of severe preeclampsia from mild preeclampsia (A:Clot angle; B:Coagulation Index, C:K time; D:Reaction time)

The TEG relevant parameters in differential diagnostic of severe preeclampsia to mild preeclampsia were demonstrated in table 3. The K time had the highest diagnostic sensitivity (96.15%), but its specificity was relative low (31.25%). The Coagulation Index had the highest specificity (0.75%), but its sensitivity was relative low (73.08%). The AUC for Clot angle, Coagulation Index, K time and Reaction time were 0.59, 0.75, 0.68 and 0.70 respectively, **Figure 4**.

**Table 3:** Efficacy of clot angle, Coagulation Index, K time and reaction time in differential diagnosis of severe preeclampsia from mild preeclampsia

Index	Sen	Spe	AUC	Cut off
Clot Angle	57.69(36.92-76.65)%	55.17(41.54-68.26)%	0.59(0.47-0.72)	72.35
Coagulation Index	73.08(52.21-88.43)%	71.88(53.25-86.25)%	0.75(0.61-0.88)	3.33
K time	96.15(80.36-99.90)%	31.25(16.12-50.01)%	0.68(0.54-0.72)	1.40
R time	76.92(56.35-91.03)%	56.25(37.66-73.64)%	0.70(0.57-0.84)	4.10

### 3.4 Platelet count and coagulation test comparison

There were no statistical difference in the aspects of platelet count and parameters relevant to coagulation test for the control, mild and sever preeclampsia groups (P>0.05), Table 4.

**Table 4:** Platelet count and coagulation test comparison of control, mild and sever preeclampsia groups (^−^ *x* ± *s* )

Groups	PT(s)	APTT (s)	TT(s)	Fib (g/L)	D-dimer (mg/L)	AT-III(%)	Platelet (×10^9^/L)
Control	10.85±0.75	26.92±2.58	14.9±0.70	4.35±0.99	1.43±0.50	97.37±14.60	238.63±63.62
mild preeclampsia	10.77±0.66	27.30±3.11	15.36±2.12	4.22±1.03	1.51±0.46	90.05±17.19	242.50±64.81
severe preeclampsia	10.57±0.62	26.15±4.40	15.51±1.10	3.96±0.99	1.68±0.72	81.85±18.97	231.23±53.21

## 4 Discussion

In recent years, the studies relevant to preeclampsia and coagulation function have become hot spots. Animal researches mainly focus on the activation and pathway of coagulation factors in preeclampsia [14]. Clinical studies of preeclampsia mainly discussed about the biomarkers for preeclampsia early diagnosis [15, 16, 17]. Previous studies have shown that hypercoagulability was more pronounced in preeclampsia patients than in normal pregnant women, but conventional coagulation tests were too slow to monitor the development of the disease [18]. TEG, as a method to simulate the dynamic process of coagulation and fibrinolysis in the human body, requires only a small amount of whole blood. Without pretreatment, TEG can monitor the interaction between coagulation factors, fibrinogen, fibrinolytic system, platelets and other cellular components, dynamically evaluate coagulation cascade reaction [19, 20, 21]. Compared to conventional coagulation tests, TEG can more comprehensively reflect the whole process of coagulation and fibrinolysis, accurately provide a profile of coagulation in patients with preeclampsia. TEG was recommended for coagulation function monitoring by The European guideline on management of major bleeding and coagulopathy following trauma to monitor coagulation function [22]. TEG is often discussed in the pregnant women with abnormal platelet function. However, clinical studies relevant to diagnostic efficacy of TEG in patients with preeclampsia and its association with blood coagulation were seldom reported in the literature.

The results of this study showed that there was no statistical difference in the conventional coagulation parameters (PT, APTT, TT, Fib, D-dimer, AT-III, PLT) between the control group, mild preeclampsia group and severe preeclampsia group, indicating that the traditional coagulation parameters could not accurate reflect the coagulation status, and the coagulation abnormal in preeclampsia could not be demonstrated by conventional coagulation parameters. The results also showed that although there was no significant difference in D-dimer, AT-III and other parameters between different groups, D-dimer increased in preeclampsia patients and AT-III decreased, indicating that with the development of preeclampsia, the risk of vascular embolism disease were increased. We also found that TEG parameters in normal pregnancy group, mild preeclampsia group and severe preeclampsia group were significantly different. This difference can be used as an important marker for diagnosing preeclampsia and evaluating the severity of the disease. TEG is superior to the traditional blood coagulation function test to a certain extent, more accurately reflects the abnormal blood coagulation function of pregnant women, especially preeclampsia patients, and provides more accurate experimental basis for monitoring blood coagulation function of preeclampsia patients. TEG can be used as a reliable monitoring index to judge the severity of preeclampsia. With the development of preeclampsia, coagulation dysfunction was more significant, breaking the dynamic balance of hypercoagulable state in normal pregnancy, and developing into a pathological hypercoagulable state, thus increasing the risk of organ thrombosis. TEG also can be used as markers to distinguish normal hypercoagulable state of pregnant women from pathological hypercoagulable state caused by preeclampsia, so as to detect abnormal coagulation function as soon as possible. To some extent, TEG can reflect the severity of preeclampsia, and can be used as an index for predicting and monitoring the condition of preeclampsia. However, TEG also had limitations such as vWD and drug related, renal failure related platelet inhibition can not be detected with TEG. Therefore, patients with the above disease was not suitable for platelet function evaluate the by TEG.

In conclusion, TEG can provide useful information in monitoring the blood coagulation of preeclampsia patients and can be used as a reliable marker for assessing the severity of preeclampsia. However, the sample size of this work was relative small with less powerful clinical evidence. Therefore, Multicenter large sample size prospective study was needed to further evaluate the diagnostic efficacy of TEG in patients preeclampsia and its association with blood coagulation.

## Abbreviations

CI: Coagulation Index
PT: Prothrombin time
APTT: activated partial thromboplastin time
Fib: fibrinogen
AT-Ⅲ: antithrombin Ⅲ
TEG: thrombelastography
ROC: receiver operating characteristic curve
AUC: area under the ROC curve
DIC: disseminated intravascular coagulation
MODS: multiple organ dysfunction syndrome

## References

[1] Piccoli GB, Cabiddu G, Castellino S, Gernone G, Santoro D, Moroni G, Spotti D, Giacchino F, Attini R, Limardo M, Maxia S, Fois A, Gammaro L, Todros T (2017). A best practice position statement on the role of the nephrologist in the prevention and follow-up of preeclampsia: the Italian study group on kidney and pregnancy. J Nephrol.

[2] Findeklee S, Costa SD, Tchaikovski SN (2015). [Thrombophilia and HELLP syndrome in pregnancy - case report and overview of the literature]. Z Geburtshilfe Neonatol.

[3] Baptista FS, MRFL B, FRM B, VLJ K, Zugaib M, RPV F (2018). Can thrombophilia worsen maternal and perinatal outcomes in cases of severe preeclampsia. Pregnancy Hypertens.

[4] DRA R, Alpoim PN, Godoi LC, Mendes FS, Lwaleed B, Sousa LP, Perucci LO, Carvalho MG, KBG B, LMS D (2017). Is there a link among thrombophilia factors and preeclampsia. J Thromb Thrombolysis.

[5] Mastrolia SA, Novack L, Thachil J, Rabinovich A, Pikovsky O, Klaitman V, Loverro G, Erez O (2016). LMWH in the prevention of preeclampsia and fetal growth restriction in women without thrombophilia. A systematic review and meta-analysis. Thromb Haemost.

[6] Sibai BM (2005). Thrombophilia and severe preeclampsia: time to screen and treat in future pregnancies. Hypertension.

[7] Mello G, Parretti E, Marozio L, Pizzi C, Lojacono A, Frusca T, Facchinetti F, Benedetto C (2005). Thrombophilia is significantly associated with severe preeclampsia: results of a large-scale, case-controlled study. Hypertension.

[8] Berks D, Duvekot JJ, Basalan H, De Maat MP, Steegers EA, Visser W (2015). Associations between phenotypes of preeclampsia and thrombophilia. Eur J Obstet Gynecol Reprod Biol.

[9] Mutoh S, Kobayashi M, Hirata J, Itoh N, Maki M, Komatsu Y, Yoshida A, Sasa H, Kuroda K, Kikuchi Y (1992). Studies of blood coagulation-fibrinolysis regarding kallikrein-kinin system in severe preeclampsia. Agents Actions Suppl.

[10] Kobayashi T, Tokunaga N, Sugimura M, Suzuki K, Kanayama N, Nishiguchi T, Terao T (1999). Coagulation/fibrinolysis disorder in patients with severe preeclampsia. Semin Thromb Hemost.

[11] Kobayashi T, Tokunaga N, Sugimura M, Kanayama N, Terao T (2001). Predictive values of coagulation/fibrinolysis parameters for the termination of pregnancy complicated by severe preeclampsia. Semin Thromb Hemost.

[12] Meier J (2012). A new application for thrombelastography in pregnant women at term. Minerva Anestesiol.

[13] Orlikowski CE, Moodley J, Rocke DA (1991). Thrombelastography in pregnant patients on low-dose aspirin. Lancet.

[14] Lwaleed BA, Dusse L, Cooper AJ (2011). Tissue factor dependent pathway and the diagnosis of pre-eclampsia. Semin Thromb Hemost.

[15] Litang Z, Hong W, Weimin Z, Xiaohui T, Qian S (2017). Serum NF-κBp65, TLR4 as Biomarker for Diagnosis of Preeclampsia. Open Med (Wars).

[16] Black C, da SCF (2018). Biomarker Immunoassays in the Diagnosis of Preeclampsia: Calculating the sFlt1/PlGF Ratio Using the Cobas®e 411 Analyser. Methods Mol Biol.

[17] Tang P, Xu J, Xie BJ, Wang QM (2017). Use of serum and urinary soluble sFlt-1 and PLGF in the diagnosis of preeclampsia. Hypertens Pregnancy.

[18] Dusse LM, Carvalho MG, Cooper AJ, Lwaleed BA (2011). Plasma factor VII: a potential marker of pre-eclampsia. Thromb Res.

[19] Parameswaran A, Krishnamoorthy VP, Oommen AT, Jasper A, Korula RJ, Nair SC, Poonnoose PM (2016). Is pre-operative assessment of coagulation profile with Thrombelastography (TEG) useful in predicting venous thromboembolism (VTE) following orthopaedic surgery. J Clin Orthop Trauma.

[20] Liu J, Wang N, Chen Y, Lu R, Ye X (2017). Thrombelastography coagulation index may be a predictor of venous thromboembolism in gynecological oncology patients. J Obstet Gynaecol Res.

[21] Lancé MD (2015). A general review of major global coagulation assays: thrombelastography, thrombin generation test and clot waveform analysis. Thromb J.

[22] Rossaint R, Bouillon B, Cerny V, Coats TJ, Duranteau J, Fernández-Mondéjar E, Filipescu D, Hunt BJ, Komadina R, Nardi G, Neugebauer EA, Ozier Y, Riddez L, Schultz A, Vincent JL, Spahn DR (2016). The European guideline on management of major bleeding and coagulopathy following trauma: fourth edition. Crit Care.

